# Type 2 diabetes and the risk of hospitalisation and mortality from seasonal influenza: an observational register-based study in Sweden

**DOI:** 10.1136/bmjopen-2025-106480

**Published:** 2026-06-22

**Authors:** Elin Allansson Kjölhede, Hanne Krage Carlsen, Oliver Martyn, Lena Svensson, Magnus Gisslén, Björn Eliasson, Katarina Eeg-Olofsson

**Affiliations:** 1 Department of Medicine, Sahlgrenska University Hospital, Gothenburg, Sweden; 2 Sahlgrenska Academy, Department of Molecular and Clinical Medicine, University of Gothenburg, Gothenburg, Sweden; 3 Centre of Registers Västra Götaland, Gothenburg, Sweden; 4 Vaccines Medical Affairs, Sanofi, Copenhagen, Denmark; 5 Vaccines Medical Affairs, Sanofi, Stockholm, Sweden; 6 Department of Infectious Diseases, Institute of Biomedicine, University of Gothenburg Sahlgrenska Academy, Gothenburg, Sweden; 7 Department of Infectious Diseases, Region Västra Götaland, Sahlgrenska University Hospital, Gothenburg, Sweden; 8 Department of Molecular and Clinical Medicine, Institute of Medicine, Sahlgrenska Academy, Gothenburg, Sweden

**Keywords:** Mortality, Diabetes Mellitus, Type 2, EPIDEMIOLOGY, Hospitalization

## Abstract

**Abstract:**

**Objectives:**

To compare influenza-related hospitalisation, mortality and effects of background factors in adults with type 2 diabetes (T2D) and age-matched and sex-matched controls. To explore associations between clinical variables and the risk of severe influenza outcomes.

**Design:**

Register-based nationwide cohort study.

**Settings:**

Data from the National Diabetes Register are cross-linked to the Swedish Population Register, Statistics Sweden and the Swedish Patient Register.

**Participants:**

371 811 patients with T2D from the Swedish National Diabetes Register and 1 728 856 matched control individuals from the Swedish population followed over the 2013/14–2018/19 influenza seasons.

**Primary and secondary outcomes:**

Data on hospital admissions, mortality, socioeconomic factors and pre-existing conditions were collected. Risk associations were analysed using Cox proportional hazards models. Within the diabetes group, non-linear associations between common clinical variables and influenza hospitalisation were examined.

**Results:**

1.6% of those with diabetes and 1.0% of controls were hospitalised for influenza, with influenza-related mortality at 0.12% and 0.08% respectively. Adjusted HR for hospitalisation in T2D was 1.57 (95% CI 1.52 to 1.61) and 1.44 (95% CI 1.29 to 1.61) for mortality. Absolute risk was highest in those with cardiovascular, kidney or respiratory disease. Relative risk was greatest in younger (<65 years) patients with T2D. Higher haemoglobin A1c (HbA1c) and lower estimated glomerular filtration rate (eGFR) were linked to increased hospitalisation risk in persons with T2D.

**Conclusion:**

This study confirms that T2D increases the risk of hospitalisation and mortality from seasonal influenza. Support to achieve HbA1c and eGFR targets and following vaccination guidelines is important.

**STRENGTHS AND LIMITATIONS OF THIS STUDY**

- A large nationwide cohort of persons with type 2 diabetes and a matched control group from the general Swedish population were followed during several influenza seasons.
- Cross-linkage of several mandatory national registers provides data on socioeconomic factors, pre-existing diagnoses, hospitalisation and mortality data.
- The Swedish National Diabetes Register provides detailed diabetes-related data from persons living with type 2 diabetes.
- Lack of information on vaccination status is a limitation in this study.

## Introduction

 In 2024, it was estimated that 587 million people worldwide had type 2 diabetes. It is assumed that the prevalence will continue to increase in coming decades.^1^ In Sweden, in 2024, more than 450 000 persons lived with type 2 diabetes.^2^

Influenza virus variants circulate in our environment and cause epidemics in populations in temperate climate zones during the colder parts of the year. The yearly epidemics cause morbidity, mortality and put strain on healthcare systems. According to Paget *et al*,^3^ approximately 389 000 deaths worldwide are attributable to influenza each year. The WHO estimates about a billion cases and between 250 000 and 650 000 deaths annually.^4^

Most persons affected by seasonal influenza experience mild symptoms but persons with underlying acute and chronic illnesses are considered at higher risk for severe outcomes when affected. A recently published meta-analysis of mostly small observational studies, many of which were case-control studies, showed that having diabetes of any type was associated with a higher risk of hospitalisation for influenza or pneumonia (adjusted OR 3.6).^5^ As in that study, most previously published studies on diabetes-related risk for hospitalisation and mortality from seasonal influenza look at all persons with diabetes as affected by the same illness. However, the pathogeneses and clinical courses of type 1 and type 2 diabetes differ significantly, and this might affect the prognosis of influenza. Current vaccination guidelines from the WHO,^4^ the Centres for Disease Control (CDC),^6^ the European Centre for Disease Control (ECDC)^7^ and the Public Health Agency of Sweden (FHM)^8^ are based on these pre-existing studies. In Sweden, persons aged 65 and older, living with chronic cardiovascular disease, chronic pulmonary disease, chronic liver or kidney failure, conditions that affect lung function or ability to cough, type 1 and type 2 diabetes and conditions affecting the immune system are recommended yearly influenza vaccinations.^8^

We have previously published data on the risk of hospitalisation with influenza in type 1 diabetes.^9^ The aim of this study was to quantify the risk of hospitalisation and mortality related to influenza in type 2 diabetes relative to a matched population without type 2 diabetes. We also wanted to evaluate the effects of age, sex, socioeconomic factors and comorbidities considered to increase the risk of influenza hospitalisation and mortality. Within the type 2 diabetes group, we wanted to investigate the association between clinical variables such as HbA1c, lipids and kidney function, routinely followed in diabetes care and risk of severe outcomes from seasonal influenza.

## Material and methods

### Population selection

In this study, we included persons with type 2 diabetes aged 18 and older from the Swedish National Diabetes Register (NDR). The NDR is a nationwide quality register for diabetes care comprising approximately 85% of persons with type 2 diabetes in Sweden.^10^ We used an epidemiological definition of type 2 diabetes used in several studies from the NDR.^11 12^ We defined type 2 diabetes as a diagnosis at the age of 40 or later regardless of diabetes treatment or treatment with diet or oral agents only, regardless of age at diagnosis. For each person with type 2 diabetes recruited into the NDR, five reference individuals without diabetes (henceforth called controls) were recruited from the Swedish Population Register into our study base. The rationale for using five reference individuals per person with diabetes instead of the whole population was to have a control population with a similar age-, sex- and socioeconomic composition as the diabetes population. The controls were sampled at random, with replacement and matched for age, sex and county of residence. ‘With replacement’ means that a control person could be sampled at random more than once and might serve as a control for more than one person with diabetes. Our study started on 1 October 2013. We excluded all persons who had died or emigrated before study start. Patients recruited into the NDR after study start were excluded. The participants in the closed cohort were followed until the end of the study in December 2019, migration or death, whichever occurred first. See online supplemental figure 4).

We defined influenza risk groups in accordance with the Swedish public health authorities’ vaccination recommendations: diabetes, age 65 years or older, presence of coronary heart disease or stroke (major acute cardiovascular events (MACEs)), chronic kidney disease (CKD) or presence of chronic lower respiratory disease.

### Outcomes

Since 2015, seasonal influenza has been a notifiable disease in Sweden. Laboratories and clinicians must report cases to the Public Health Agency of Sweden. In this study, data on influenza-related hospitalisation, hospitalisation duration and mortality were obtained from the mandatory Swedish National Patient Register and the Swedish Cause of Death Register using the International Classification of Diseases -10 codes J09.9 and J10. We defined influenza season as calendar week 40–week 20 the following year (October–May), as this is the period where persons in Sweden are under high risk of influenza infection in concordance with the vigilance period defined by the Public Health Agency of Sweden. Only influenza admissions during these periods were included in the analyses. One, the first of several, hospital admission for influenza per season per person was registered. We defined influenza-related mortality as death within 28 days of influenza hospitalisation, a commonly used metric when studying mortality risk from infectious disease. It balances the need to include deaths due to secondary infections and complications and the risk of overestimating mortality risk when extending the observation period.

### Covariates included and analysed in the study

We collected data on pre-existing diagnoses from the Swedish National Patient Register for both persons with type 2 diabetes and controls from 2005 until study start. The mandatory Swedish National Patient Register gathers ICD codes from all hospital stays and outpatient visits to specialised clinics. Pre-existing conditions were coronary heart disease, stroke, heart failure, atrial fibrillation, chronic pulmonary disease, kidney failure and cancer. For a full list of included diagnoses and ICD codes, see online supplemental table 2).

Information about socioeconomic status indicators, including marital status, country of birth, income and education, were collected at the study baseline year from the Longitudinal Integrated Database for Health Insurance and Labour Market Studies (LISA), Statistics Sweden.

We collected clinical characteristics concerning persons with type 2 diabetes and information on antihyperglycaemic, antihypertensive and lipid-lowering treatment from the NDR. We estimated metabolic control using glycated haemoglobin (HbA1c, mmol/mol). Furthermore, we collected data on body mass index (BMI), defined as body mass (kg) divided by the square of body height (kg/m^2^); kidney function, measured as estimated glomerular filtration rate (eGFR, mL/min/1.73 m^2^); total cholesterol, high-density lipoprotein cholesterol (HDL-C); low-density lipoprotein cholesterol (LDL-C) and triglycerides (TGs). Registered blood pressure measurements and lifestyle factors, such as smoking habits and level of physical activity, were included in the study. These clinical characteristics were collected within 1 year of study start, apart from smoking habits and level of physical activity, where the last observation was carried forward.

Information on contributing factors to mortality was gathered from the Swedish Cause of Death register.

### Statistical methods

Descriptive data concerning persons with diabetes and controls are presented in table 1. We also present data separated by hospitalisation status in the diabetes group. The cumulative incidence of influenza hospitalisation and influenza-related mortality was estimated in individuals with diabetes and controls using a Kaplan-Meier estimator. We calculated incidence rates (cases/10,000 person-years) in the different groups, including risk groups with a high risk of influenza complications. We used Cox proportional hazard models to compare risk for influenza hospitalisation and influenza-related mortality in risk group strata adjusted for age and sex, when appropriate. Associations between diabetes and influenza outcomes compared with controls were quantified using Cox proportional hazards models. We tested the importance of socioeconomic variables and pre-existing comorbidities on influenza risk in type 2 diabetes by adding them stepwise to the models. Additional analyses were performed in persons with diabetes as more data on clinically relevant variables were available. For numerical clinical variables, Cox regression with splines (with four df) was used to investigate non-linear associations with influenza hospitalisation risk.

**Table 1:** Baseline characteristics of the cohort

	Type 2 diabetes, influenza (n=5871)	Type 2 diabetes, no influenza (n=3 65 940)	Type 2 diabetes, all (n=371 811)	Controls (n=1 728 856)
Sex, female	2599 (44.3%)	161 105 (44.0%)	163 704 (44.0%)	764 101 (44.2%)
Age at study start, *years*	73.8 (10.0)	68.9 (12.0)	69.0 (12.0)	68.8 (12.0)
Age category at study start, years				
18–44	55 (0.9%)	10 860 (3.0%)	10 915 (2.9%)	50 477 (2.9%)
45–54	205 (3.5%)	32 926 (9.0%)	33 131 (8.9%)	158 130 (9.1%)
55–64	679 (11.6%)	75 931 (20.7%)	76 609 (20.6%)	362 981 (21.0%)
65–74	1890 (32.2%)	124 319 (34.0%)	126 207 (33.9%)	586 392 (33.9%)
75+	3042 (51.8%)	121 904 (33.3%)	124 945 (33.6%)	570 875 (33.0%)
Marital status				
Married	2915 (49.7%)	188 089 (51.4%)	191 004 (51.4%)	906 815 (52.5%)
Separated	1075 (18.3%)	64 687 (17.7%)	65 762 (17.7%)	304 323 (17.6%)
Widowed	1168 (19.9%)	56 386 (15.4%)	57 554 (15.5%)	260 912 (15.1%)
Single	709 (12.1%)	55 965 (15.3%)	56 674 (15.2%)	243 489 (14.1%)
Income quartile				
First quartile	1739 (29.6%)	94 065 (25.7%)	95 804 (25.8%)	359 687 (20.8%)
Second quartile	2051 (34.9%)	97 435 (26.6%)	99 486 (26.8%)	390 484 (22.6%)
Third quartile	1424 (24.3%)	94 744 (25.9%)	96 168 (25.9%)	455 300 (26.3%)
Fourth quartile	654 (11.1%)	79 535 (21.7%)	80 189 (21.6%)	520 691 (30.1%)
Education, years				
9 years	2703 (46.0%)	142 515 (38.9%)	145 218 (39.1%)	567 731 (32.8%)
9–12 years	2223 (37.9%)	152 368 (41.6%)	154 591 (41.6%)	704 859 (40.8%)
>12 years	766 (13.0%)	64 377 (17.6%)	65 143 (17.5%)	434 128 (25.1%)
Birth country				
Sweden	4634 (78.9%)	294 655 (80.5%)	299 289 (80.5%)	1 496 944 (86.6%)
Europe except Sweden	373 (6.4%)	19 878 (5.4%)	20 251 (5.4%)	79 996 (4.6%)
Rest of the world	864 (14.7%)	51 407 (14.0%)	52 271 (14.1%)	151 916 (8.8%)
Previous disease				
Pneumonia (J12-18)	252 (4.3%)	9641 (2.6%)	9893 (2.7%)	39 944 (2.3%)
Chronic lower respiratory disease (J40-47)	550 (9.4%)	18 058 (4.9%)	18 608 (5.0%)	69 352 (4.0%)
Kidney failure (N17-9)	216 (3.7%)	6596 (1.8%)	6812 (1.8%)	13 966 (0.8%)
Stroke (I61-I64)	421 (7.2%)	16 915 (4.6%)	17 336 (4.7%)	58 354 (3.4%)
Coronary heart disease (CHD) (I20-I21)	1361 (23.2%)	59 192 (16.2%)	60 553 (16.3%)	160 205 (9.3%)
Cardiovascular (CVS, I21-25)	868 (14.8%)	37 325 (10.2%)	38 193 (10.3%)	100 516 (5.8%)
Atrial fibrillation (I48)	813 (13.8%)	29 630 (8.1%)	30 443 (8.2%)	106 928 (6.2%)
Heart failure (I50)	596 (10.2%)	20 067 (5.5%)	20 663 (5.6%)	51 065 (3.0%)
Cancer (C)	917 (15.6%)	48 618 (13.3%)	49 535 (13.3%)	240 530 (13.9%)
MACE (I20-I21, I61-64, I48, I50)	2584 (44.0%)	106 359 (29.1%)	108 943 (29.3%)	323 699 (18.7%)
Diabetes duration, years	11.0 (8.1)	9.4 (7.3)	9.5 (7.4)	
Weight, kg	85.9 (18.4)	86.3 (18.0)	86.3 (17.9)	
Height, cm	169.1 (11.0)	170.2 (10.2)	170.2 (10.2)	
Body mass index, kg/m^2^	29.8 (5.4)	29.6 (5.3)	29.6 (5.3)	
HbA1c, mmol/mol	56.7 (14.7)	54.1 (13.5)	54.1 (13.5)	
Systolic blood pressure, mm Hg	135.5 (16.6)	135.1 (15.7)	135.1 (15.7)	
Diastolic blood pressure, mm Hg	74.7 (9.9)	76.3 (9.7)	76.2 (9.7)	
HDL cholesterol, mmol/L	1.3 (0.4)	1.3 (0.4)	1.3 (0.4)	
LDL cholesterol, mmol/L	2.6 (1.0)	2.7 (0.9)	2.7 (0.9)	
Total cholesterol, mmol/L	4.6 (1.2)	4.7 (1.1)	4.7 (1.1)	
Triglycerides, mmol/L	1.8 (1.0)	1.8 (1.2)	1.8 (1.2)	
Estimated glomerular filtration rate	72.6 (25.9)	79.8 (24.5)	79.7 (24.6)	
Creatinine, mg/mmol	91.0 (45.7)	82.0 (34.2)	82.1 (34.4)	
Albuminuria				
Macroalbuminuria	234 (11.6%)	9953 (7.3%)	10 187 (7.4%)	
Microalbuminuria	481 (23.8%)	24 275 (17.9%)	24 756 (18.0%)	
Lipid-lowering treatment	2273 (38.7%)	139 848 (38.2%)	142 121 (38.2%)	
Antihypertensive medication	3045 (51.9%)	175 592 (48.0%)	178 637 (48.0%)	
Antihyperglycaemic treatment				
Diet only	1313 (23.6%)	86 291 (24.7%)	87 604 (24.7%)	
Insulin only	999 (18.0%)	43,0338 (12.3%)	44 032 (12.4%)	
Oral agent (incl. Glucagon-Like Peptide-1)	2231 (40.1%)	167 920 (48.1%)	170 151 (47.9%)	
Oral agent and insulin	1014 (18.2%)	52 205 (14.9%)	53 219 (15.0%)	
Lifestyle factors				
Smoker	753 (12.8%)	47 548 (13.0%)	48 301 (13.0%)	
Physical activity				
Exercise 1–5 times per week	1873 (39.5%)	130 335 (43.6%)	132 208 (43.5%)	
Exercise daily	1072 (22.6%)	84 708 (28.3%)	85 780 (28.2%)	
Exercise never or rarely	1797 (37.9%)	84 126 (28.1%)	85 923 (28.3%)	

Continuous variables are shown as mean (SD), and categorical variables are shown as number (proportion) of the total.

AF, atrial fibrillation; BMI, body mass index; CHD, coronary heart disease; CVS, cardiovascular; HbA1c, haemoglobin A1c; HDL, high-density lipoprotein; LDL, low-density lipoprotein; MACE, major adverse cardiovascular event.

Less than 2% of the study population had missing data on pre-existing comorbidities from the patient register and on socioeconomic status from Statistics Sweden.

Statistical analyses were performed using R (R Foundation for Statistical Computing, Vienna, Austria; http://www.R-project.org), V.4.0. The registration of healthcare data in administrative registers and its use in research and for other purposes is regulated by Swedish law and not subject to informed consent (Patient Data Act 2008:355, chapter 7). The Swedish Ethical Review Authority approved this study, dnr 2019-00899.

### Patient and public involvement

Patients and/or the public were not involved in the design, conduct, report and dissemination plans of this research.

## Results

### Descriptive statistics

Our closed cohort included 371 811 individuals with type 2 diabetes and 1 728 856 controls. 44% were female, and the mean age at study start was 69 years. 80.5% of the diabetes group and 86.6% of controls were born in Sweden. The proportion of persons in the highest income and education quartiles was higher in the control group. Marital status was similar between the groups. Pre-existing diagnoses of coronary heart disease, atrial fibrillation, heart failure, chronic respiratory disease and kidney failure were more common among persons with type 2 diabetes. However, cancer did not differ between the groups. Information on socioeconomic factors and pre-existing medical conditions as well as anthropometric data, data on metabolic control, cardiovascular risk factors and medication in the type 2 diabetes group is presented in table 1.

### Influenza hospitalisation

**Figure 1:**
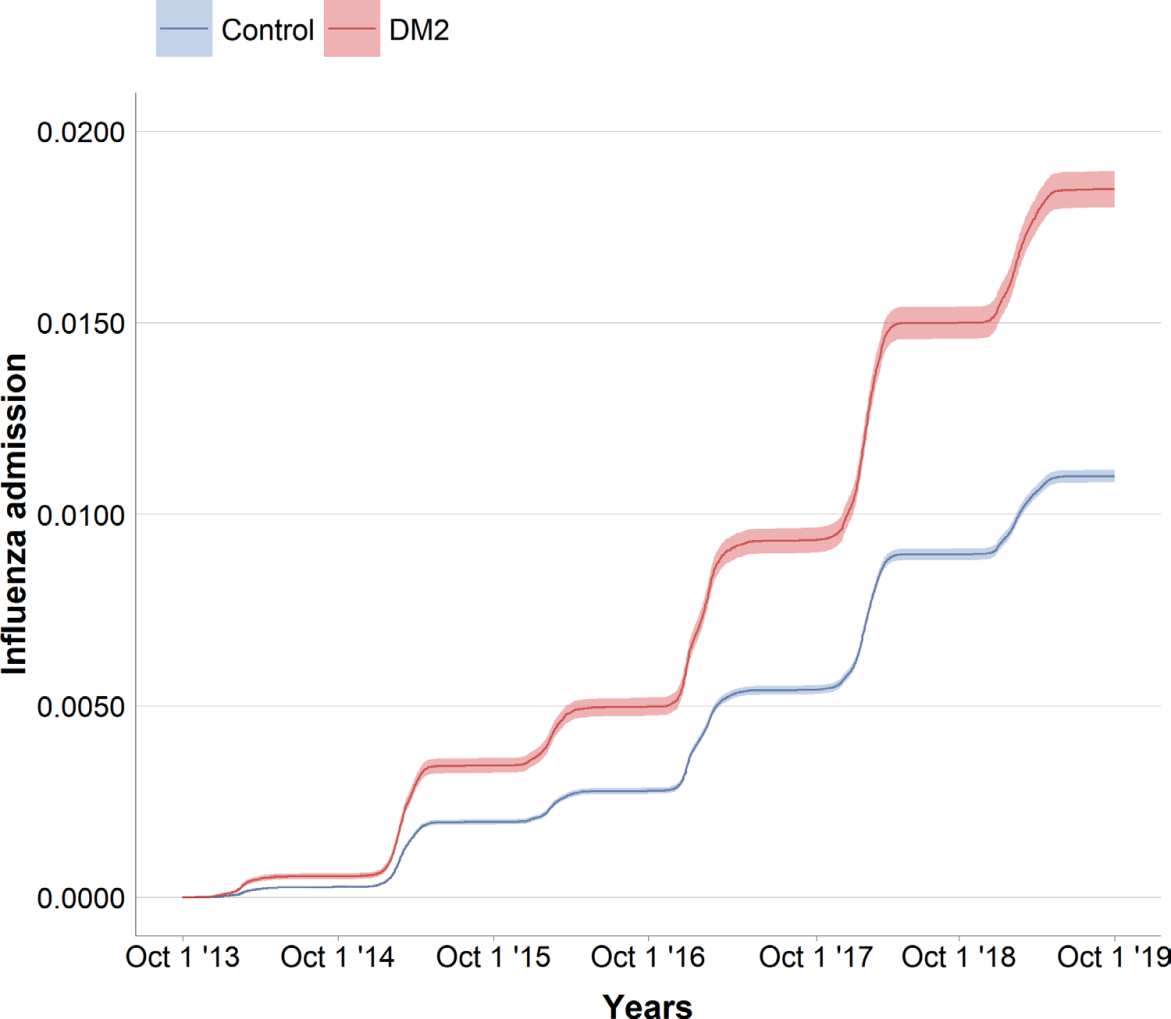
Kaplan-Meier plot of influenza admission. DM2, Type 2 diabetes

**Figure 2:**
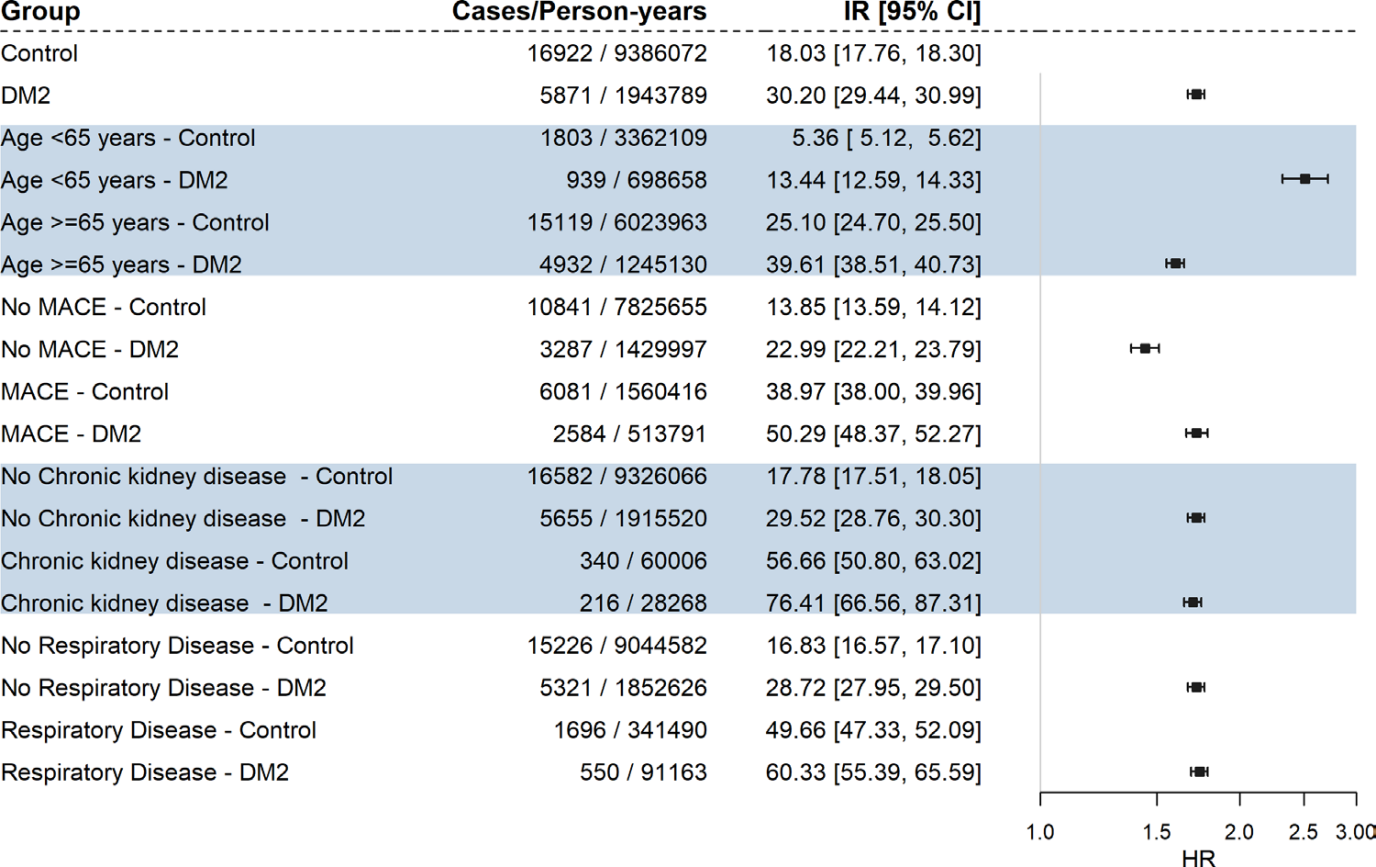
Number of events, incidence rates and HRs associated with type 2 diabetes compared with controls for influenza hospitalisation in risk group strata based on age and pre-existing conditions. CKD, chronic kidney disease; DM2, Type 2 diabetes, HR, hazard ratio associated with type 2 diabetes, obtained from stratified models adjusted for age and sex; IR, incidence rate per 10 000 person-years; MACEs: major adverse cardiovascular events (coronary heart disease, stroke, heart failure or atrial fibrillation); RD, respiratory disease (chronic obstructive pulmonary disease).

In the diabetes group, 5871 (1.6%) were hospitalised with influenza during the study period with a mean follow-up time of 5.4 years. The corresponding number in the control group was 16 992 (1.0%). The mean hospitalisation duration was 8 days in both groups (see online supplemental table 1). Cumulative incidence of influenza hospitalisation over the study period, influenza seasons 2013/14–2018/19, is shown in figure 1. In figure 2, we present incidence rates and HRs for hospitalisation in different subgroups, where results are adjusted for age and sex. The incidence rate per 10 000 person-years for influenza hospitalisation was 30.2 in the diabetes group and 18.0 in the control group. We stratified the data by age under or over 65. In the group under the age of 65 years, the incidence rate was 13.4 per 10 000 person-years and 5.4 among controls. In those 65 and older, the incidence rate was 39.6 and 25.1, respectively. Sub-grouping the data based on pre-existing major adverse cardiovascular events (MACE) gave an incidence rate of 50.3 in persons with type 2 diabetes compared with 39.0 in controls. In CKD, the incidence rate was 76.4 in type 2 diabetes and 56.7 in controls.

Overall, in the diabetes group, the HR for influenza-related hospital admission was 1.7. We then stratified the analyses according to risk groups recommended for yearly vaccinations by the Public Health Agency of Sweden. In those under the age of 65, the HR was 2.5 for hospitalisation for the diabetes groups. The corresponding number of persons over 65 was 1.6. In persons with pre-existing MACE the HR for hospitalisation was 1.7.

**Figure 3:**
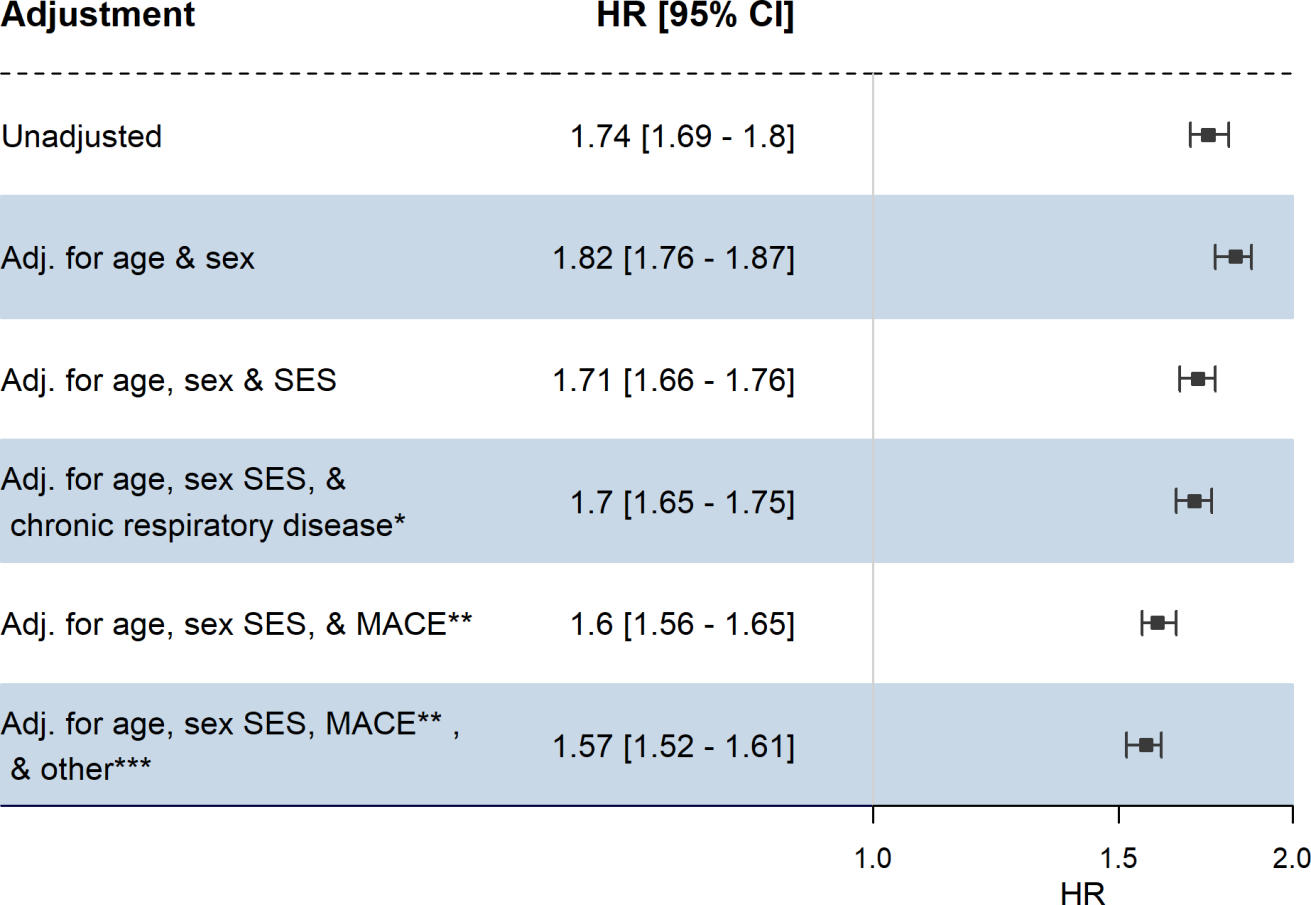
Unadjusted and adjusted HRs of influenza hospitalisations in persons with type 2 diabetes compared with controls at different levels of adjustment age: age at baseline. SES, socioeconomic status: composite of education, income, birth region and marital status. *Chronic respiratory disease (chronic obstructive respiratory disease). ** MACE, major adverse cardiovascular event (coronary heart disease, stroke, heart failure or atrial fibrillation). *** Cancer, kidney disease.

To analyse the importance of socioeconomic variables and pre-existing comorbidities on influenza hospitalisation risk in type 2 diabetes we added the covariates stepwise to the Cox models and found an HR of 1.74 in the crude model for influenza-related hospitalisation in persons with type 2 diabetes in comparison to controls, HR 1.71 with adjustment for age, sex and socioeconomic status and HR 1.57 for the fully adjusted model. All adjustment levels can be seen in figure 3.

### Influenza-related mortality

Among those hospitalised with influenza, 429 (0.12%) persons with type 2 diabetes and 1316 (0.08%) of the control group died within 28 days. Cumulative incidence of influenza-related mortality over the study period can be seen in online supplemental figure 1). During the study, non-influenza-related death was higher in the type 2 diabetes group compared with the controls, 23.6% and 17.6%, respectively (see online supplemental table 1). The incidence rate for influenza-related death per 10 000 person-years was 1.4 in controls compared with 2.2 in persons with type 2 diabetes. Among those aged 65 and older, the incidence rate was 2.1 in controls and 3.1 in type 2 diabetes. In persons younger than 65 years, controls had an incidence rate of 0.2 compared with 0.5 in the diabetes group. Further data are shown in online supplemental figure 2). Overall, the HR for influenza-related mortality was 1.6. We stratified the analyses according to risk groups defined by the Swedish Public Health Agency for influenza-related mortality and adjusted for age, sex and socioeconomic status. The group under the age of 65 had an HR of 2.7 (95% CI 1.8 to 4.1), whereas those 65 and older had an HR of 1.5 (95% CI 1.4 to 1.7). In the strata with/without MACE, the HR was 1.7 (95% CI 1.4 to 1.9) and 1.4 (95% CI 1.2 to 1.6), respectively. In the strata with/without CKD, the HR was 1.6 (95% CI 1.5 to 1.8) and 1.6 (95% CI 1.5 to 1.8), respectively. In the strata with/without chronic respiratory disease, the HR for mortality was 1.6 (95% CI 1.5 to 1.8) and 1.6 (95% CI 1.5 to 1.8), respectively.

Adjusting the risk model for socioeconomic factors and pre-existing comorbidities attenuated the HR as associated with diabetes from an HR of 1.58 (unadjusted) to HR of 1.44 (fully adjusted) for influenza-related mortality in persons with type 2 diabetes compared with controls. See online supplemental figure 3.

Using the Swedish Cause of Death Register, we studied the primary contributing causes of death among individuals who passed away within 28 days of hospitalisation due to influenza. The most common primary causes of death were chronic pulmonary disease, pneumonia and seasonal influenza. Seasonal influenza and heart disease, predominantly ischaemic heart disease, along with endocrine disorders, were prevalent contributing factors. The observed pattern was similar in both individuals with type 2 diabetes and control subjects.

### Influenza hospitalisation and clinical covariates in type 2 diabetes

Within the type 2 diabetes group, we analysed associations between clinical covariates, systolic and diastolic blood pressure, eGFR, lipid levels and HbA1c, and the risk of influenza hospital admission using splines (see online supplemental figure 5 a–i. Increased risk of hospitalisation was associated with higher HbA1c and lower eGFR. BMI between 20 and 45 was not associated with higher risk. TGs did not seem to affect the hospitalisation risk, whereas lower HDL-C was associated with a higher risk of hospitalisation. LDL-C levels did not show a clear association with risk of hospitalisation.

## Discussion

Our extensive nationwide study delves into the risk of influenza-related hospitalisation and mortality within a cohort comprising 371 811 individuals with type 2 diabetes and 1 728 586 age- and gender-matched controls. Over six influenza seasons and during a mean follow-up time of 5.4 years, 1.6% of persons with diabetes and 1% of controls were hospitalised with influenza, and of those, 429 and 1316, respectively, died within 28 days of hospitalisation. Persons with type 2 diabetes had a 70% higher relative risk for influenza hospitalisation compared with controls, a risk that was attenuated but still 60% higher when adjusted also for socioeconomic factors and pre-existing comorbidities. For influenza-related mortality, the corresponding numbers were 60% and 40% higher relative risks in the diabetes group compared with controls. This attenuation indicates that previous illnesses as well as socioeconomic status contribute substantially to the risk of influenza-related hospitalisation and mortality in the type 2 diabetes group, perhaps due to both risk caused by the underlying illness as such and the combination with diabetes. We can only speculate which socioeconomic factors might influence the risk of severe illness. Perhaps the lower education level and the fact that a higher rate of persons with type 2 diabetes were born outside of Europe might hinder contact with healthcare units, perhaps worsening symptoms and delaying treatment. The remaining still-significant adjusted HRs likely represent the biological effects on the immune system previously shown in persons living with diabetes.

In the fully adjusted models, a history of heart failure, kidney disease and chronic respiratory disease with negative outcomes from influenza.

The absolute risk was highest among those suffering from diabetes and concurrent CKD, major adverse cardiovascular events or chronic respiratory disease. Though the absolute risk was lower in the age group under 65 years of age, the highest excess risk was seen in this group with more than a twofold higher risk for severe influenza outcomes in the diabetes group compared with controls.

compared with Hopkins *et al*,^13^ who in their study from the United Kingdom found that approximately 0.55% of their type 2 diabetes cohort was hospitalised with influenza, we found that 1.6% of the type 2 diabetes cohort was hospitalised. The studies were conducted at approximately the same time, and the same virus variants ought to have circulated in the studied populations. One possible explanation for this difference might be differences in healthcare system organisations, where the UK system had more advanced treatment options at home than the Swedish system during the studied period. Another possible explanation could be that while both these studies are register-based studies, differences could occur in the recording of diagnoses, both regarding diabetes and the outcomes in the studies.

Our findings on increased relative risk are concurrent with the findings in the meta-analysis by Dicembrini *et al*^5^ where both unadjusted (5.08) and adjusted (3.62) ORs showed an increased risk for influenza-related hospitalisation in persons with diabetes. In a US-based study by Harding *et al*^14^ age-adjusted RR for influenza hospitalisation varied from 2.2 to 2.6 in the years 2000–2015. The same pattern was seen in the study conducted in Norway by Ruiz *et al*^15^ during the pandemic influenza in 2009. All these findings show an increased risk of hospitalisation from influenza in persons with diabetes, although the magnitude of risk increase varies between included studies due to statistical methods, setting and clinical practices. Factors to consider are variations in admission criteria in different countries and changes in healthcare systems over time. For example, Luk *et al*^16^ showed increasing influenza-related hospitalisation rates in Hong Kong over the period 2001–2016. These variations could affect the rates of hospitalisation for influenza observed in different populations.

One explanation of the variation in mortality is that the definition of influenza-related death varies between different studies. In our study, we found an HR of 1.44 for influenza-related mortality after adjustments for age, gender and socioeconomic factors and other co-existing diseases. In our study, the absolute risk was higher in older individuals, but the relative risk was higher in younger individuals with type 2 diabetes. This is probably due to increased frailty and higher likelihood of pre-existing diseases in both the general population and the group with type 2 diabetes with increasing age. The higher relative risk for influenza mortality observed in younger individuals with type 2 diabetes seen in this study is in alignment with the increased health burden seen in patients with type 2 diabetes in comparison with their healthy counterparts.^17 18^

Regrettably, at the time of data collection, Swedish vaccination records were not yet digitalised or centrally collected. Therefore, we are unable to assess the impact of vaccination on the results in our study. Interpretations of the results should be made bearing this in mind. The influenza vaccination uptake in Sweden is, despite yearly vaccination campaigns, quite low. According to a report published by the Swedish public health agency in 2020, vaccination uptake in persons over the age of 65 has varied between 44% and 55% in the time period between 2010 and 2020. In persons under 65, including risk groups, vaccine uptake varies around 3% of the population.^19^ This might in part explain the higher relative risk for influenza hospitalisation seen in younger individuals in our study. Internationally, studies have found vaccination uptake to be higher in persons with type 2 diabetes and other chronic illnesses such as CKD and MACE.^20^ This might lead to an underestimation of differences in risk between groups in our study. In future studies, we hope to include vaccination data to better understand factors explaining the increased risk of hospitalisation and mortality from influenza in persons with type 2 diabetes compared with controls.

Our study showed an increased risk of hospitalisation with worse glycaemic control measured with HbA1c. The accelerating hospitalisation risk with increasing HbA1c could be explained by the immunosuppressive effect of high glucose shown by Rayfield *et al*.^21^ This effect is mediated by, among other factors, downgraded degranulation in neutrophils,^22^ impaired phagocytosis^23^ and affected complement activity.^24^ Another factor is the increased viral replication seen in hyperglycaemia^25^ leading to higher viral load and higher risk of severe infection. Our findings contradict the study published by Luk *et al*^26^ in 2017, where they found no increased risk of hospitalisation from respiratory infections with higher HbA1c.

### Strengths and limitations

The strengths of this nationwide study are that it includes most persons with type 2 diabetes in Sweden and uses validated national health registers for information on socioeconomic status, pre-existing comorbidities and outcomes in both the diabetes and the control groups. In the diabetes group, we also have access to clinical data on risk factors in the NDR. There are also limitations to the study. We only included hospital discharge diagnoses, which could underestimate the severe influenza-related events but most likely to the same extent in both the diabetes and control groups. Another obvious limitation is the lack of information on vaccination status. Hopefully, this study highlights this important area and points to the need for a national register for vaccinations.

## Conclusion

Type 2 diabetes was associated with a substantially increased risk of both hospitalisation and mortality related to seasonal influenza. This excess risk persisted after adjustment for age, sex, socioeconomic status and comorbid conditions, suggesting that type 2 diabetes itself may represent an independent risk factor. These findings support adherence to current influenza vaccination recommendations in this population. In addition, the observed association between higher HbA1c levels and an increased likelihood of hospital admission highlights the potential importance of optimising glycaemic control. Preserved renal function also appeared to be associated with a lower risk, suggesting that maintaining kidney health may represent an important component of comprehensive diabetes management.

## Supplementary material

10.1136/bmjopen-2025-106480 online supplemental file 1

## Data Availability

Data are available upon reasonable request.

## References

[1] Genitsaridi I, Salpea P, Salim A (2026). 11th edition of the IDF Diabetes Atlas: global, regional, and national diabetes prevalence estimates for 2024 and projections for 2050. Lancet Diabetes Endocrinol.

[2] NDR (2025). Swedish national diabetes register annual report.

[3] Paget J, Spreeuwenberg P, Charu V (2019). Global mortality associated with seasonal influenza epidemics: New burden estimates and predictors from the GLaMOR Project. J Glob Health.

[4] (2023). Organization WH. https://www.who.int/news-room/fact-sheets/detail/influenza-(seasonal).

[5] Dicembrini I, Silverii GA, Clerico A (2023). Influenza: Diabetes as a risk factor for severe related-outcomes and the effectiveness of vaccination in diabetic population. A meta-analysis of observational studies. Nutr Metab Cardiovasc Dis.

[6] CDC. https://www.cdc.gov/flu/professionals/acip/summary/summary-recommendations.htm.

[7] ECDC. https://www.ecdc.europa.eu/en/seasonal-influenza/prevention-and-control/vaccines/risk-groups.

[8] Sweden PHAo (2023). Vaccination against flu and covid-19. https://www.folkhalsomyndigheten.se/the-public-health-agency-of-sweden/communicable-disease-control/vaccinations/vaccination-against-flu-and-covid-19/.

[9] Kjölhede EA, Carlsen HK, Martyn O (2025). Hospitalisation from seasonal influenza among persons with type 1 diabetes: a cohort study from the Swedish National Diabetes Register. BMJ Open.

[10] Register TSND Årsrapport 2023 års resultat 2024. https://registercentrum.blob.core.windows.net/ndr/r/-rsrapport-Nationella-Diabetesregistret-2023-gCM79vAxQ.pdf.

[11] Eeg-Olofsson K, Cederholm J, Nilsson PM (2010). New aspects of HbA1c as a risk factor for cardiovascular diseases in type 2 diabetes: an observational study from the Swedish National Diabetes Register (NDR). J Intern Med.

[12] Lind M, Olsson M, Rosengren A (2012). The relationship between glycaemic control and heart failure in 83,021 patients with type 2 diabetes. Diabetologia.

[13] Hopkins R, Young KG, Thomas NJ (2024). Risk factor associations for severe COVID-19, influenza and pneumonia in people with diabetes to inform future pandemic preparations: UK population-based cohort study. BMJ Open.

[14] Harding JL, Benoit SR, Gregg EW (2020). Trends in Rates of Infections Requiring Hospitalization Among Adults With Versus Without Diabetes in the U.S., 2000–2015. Diabetes Care.

[15] Ruiz PLD, Bakken IJ, Håberg SE (2020). Higher frequency of hospitalization but lower relative mortality for pandemic influenza in people with type 2 diabetes. J Intern Med.

[16] Luk AOY, Wu H, Lau ESH (2021). Temporal trends in rates of infection-related hospitalisations in Hong Kong people with and without diabetes, 2001-2016: a retrospective study. Diabetologia.

[17] Sattar N, Rawshani A, Franzén S (2019). Age at Diagnosis of Type 2 Diabetes Mellitus and Associations With Cardiovascular and Mortality Risks. Circulation.

[18] Kaptoge S, Seshasai SRK, Sun L (2023). Life expectancy associated with different ages at diagnosis of type 2 diabetes in high-income countries: 23 million person-years of observation. Lancet Diabetes Endocrinol.

[19] Sweden PHAo (2020). Influenza in sweden - season 2019-2020.

[20] Verket M, Jacobsen M, Schütt K (2023). Influenza vaccination in patients affected by diabetes. Eur Heart J.

[21] Rayfield EJ, Ault MJ, Keusch GT (1982). Infection and diabetes: the case for glucose control. Am J Med.

[22] Stegenga ME, van der Crabben SN, Blümer RME (2008). Hyperglycemia enhances coagulation and reduces neutrophil degranulation, whereas hyperinsulinemia inhibits fibrinolysis during human endotoxemia. Blood.

[23] Alexiewicz JM, Kumar D, Smogorzewski M (1995). Polymorphonuclear leukocytes in non-insulin-dependent diabetes mellitus: abnormalities in metabolism and function. Ann Intern Med.

[24] Hair PS, Echague CG, Rohn RD (2012). Hyperglycemic conditions inhibit C3-mediated immunologic control of Staphylococcus aureus. J Transl Med.

[25] Kohio HP, Adamson AL (2013). Glycolytic control of vacuolar-type ATPase activity: a mechanism to regulate influenza viral infection. Virology.

[26] Luk AOY, Lau ESH, Cheung KKT (2017). Glycaemia control and the risk of hospitalisation for infection in patients with type 2 diabetes: Hong Kong Diabetes Registry. Diabetes Metab Res Rev.

