# Supplementary material for: Type 2 diabetes and the risk of hospitalisation and mortality from seasonal influenza: an observational register-based study in Sweden
Source: BMJ Open. 2026 Jun 22;16(6):e106480. doi: 10.1136/bmjopen-2025-106480 (PMC13288867; doi:10.1136/bmjopen-2025-106480)
Supplement: online supplemental file 1 [file bmjopen-16-6-s001.docx]

**Supplementary material**

**Supplementary table 1– Overview of outcomes in the cohort**

|  | **Type 2 diabetes (N=371,811)** | **Controls (N=1,728,856)** | **All participants (N=2,100, 667)** |
| --- | --- | --- | --- |
| Influenza admissions | 5,871 (1·6%) | 16,922 (1·0%) | 22,793 (1·1%) |
| Influenza-related death | 429 (0·12%) | 1,316(0·08%) | 1,745 (0·08%) |
| Non-influenza death | 87,752 (23·6%) | 304,102 (17·6%) | 391,934 (18·7%) |
| Alive at end of follow-up | 277,422 (74·6%) | 1,619,390 (81·2%) | 1,896,812 (80·2%) |
| Lost to follow-up (emigrated) | 766 (0·2%) | 4,826 (0·3%) | 5,592 (0·3%) |
| Follow-up time*, years* | 5·2 (1·6) | 5·4 (1·4) | 5·4 (1·4) |
| Duration of admission, *days* | 8·0 (8·0) | 7·9 (8·3) | 7·9 (8·2) |

Continuous variables shown as mean (standard deviation) and categorical variables shown as number (proportion) of the total.

**Supplementary table 2 - ICD-10 Codes used in data collection**

| Variable | ICDcode/s | Source |
| --- | --- | --- |
| Type 2 Diabetes | E11’ | Swedish National Diabetes Register |
| Influenza | J10-11, J12.9 | Swedish National Patient Register |
| Pneumonia | J12-18 | Swedish National Patient Register |
| Chronic Lower Respiratory disease | J40-47 | Swedish National Patient Register |
| Kidney failure | N17-19 | Swedish National Patient Register |
| Stroke | I61-I64 | Swedish National Patient Register |
| Acute Myocardial infarction (AMI) | I21 | Swedish National Patient Register |
| Coronary heart disease | I20-I21 | Swedish National Patient Register |
| Atrial fibrillation | I48 | Swedish National Patient Register |
| Heart failure | I50 | Swedish National Patient Register |
| Cancer | C’ | Swedish National Patient Register |
| Major adverse cardiovascular events (MACE) | I20-I21, I61-64, I48, I50 | Swedish National Patient Register |

**Supplementary figure 1 – Kaplan-Meier plot of influenza-related mortality**


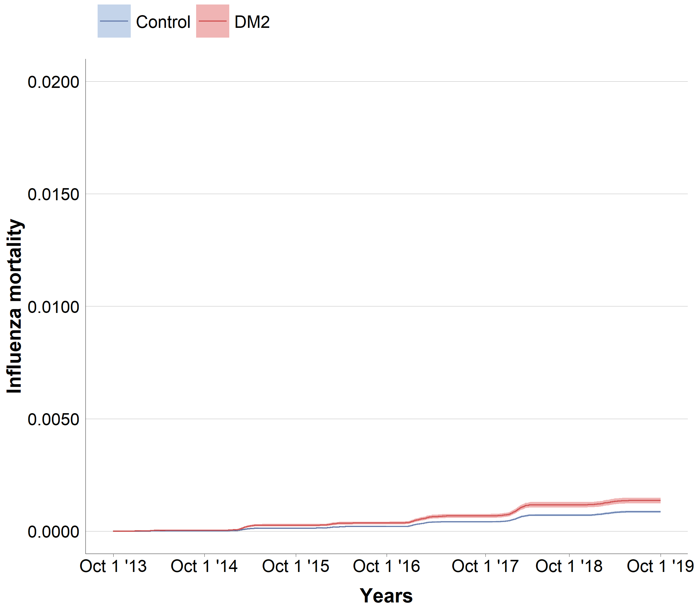


**Supplementary figure 2. Unadjusted and adjusted Hazard ratios (HRs) of influenza-related mortality in persons with type 2 diabetes compared to controls at different levels of adjustment.**

##
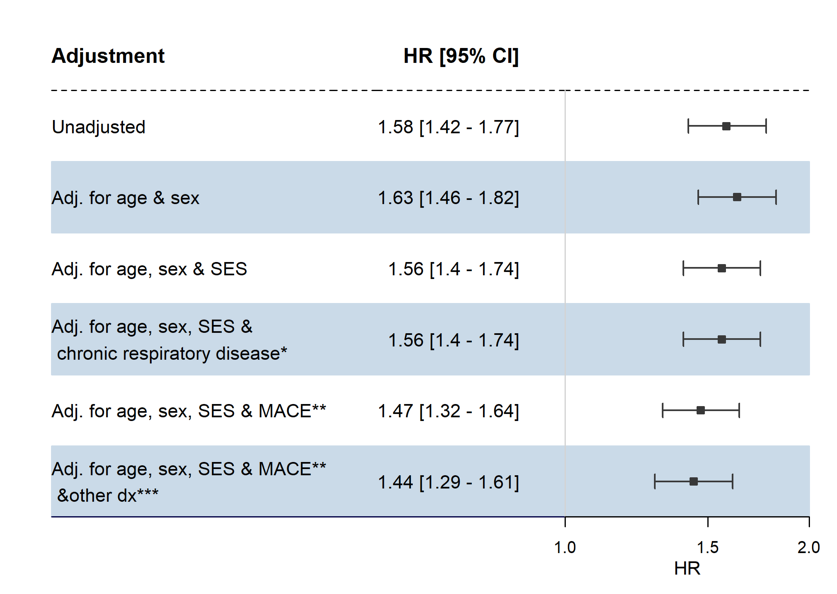


Influenza-related mortality: death within 28 days of influenza-related hospital admission. Age: Age at baseline. SES: Socioeconomic status: composite of education, income, birth region and marital status.
*Chronic respiratory disease (chronic obstructive respiratory disease)
** MACE: Major adverse cardiovascular event + (coronary heart disease, stroke, heart failure, or atrial fibrillation)
*** Cancer, kidney disease

**Supplement figure 3. Number of events, incidence rates and hazard ratios associated with type 2 diabetes compared to controls for influenza-related mortality in risk group strata based on age or pre-existing conditions**
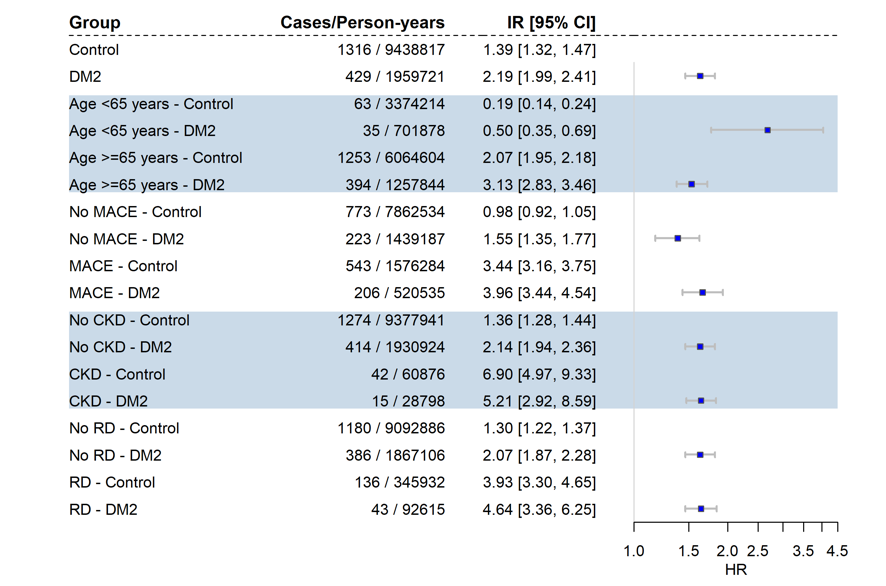


Influenza-related mortality: death within 28 days of an influenza-related hospital admission. IR: Incidence rate per 10 000 person-years. HR: Hazard Ratio associated with type 2 diabetes, obtained from stratified models adjusted for age and sex. MACE: Major adverse cardiovascular event + (coronary heart disease, stroke, heart failure, or atrial fibrillation). CKD: Chronic kidney disease. RD: Respiratory disease (chronic obstructive pulmonary disease)

**Supplementary figure 4 – Flowchart – overview of the data selection process**


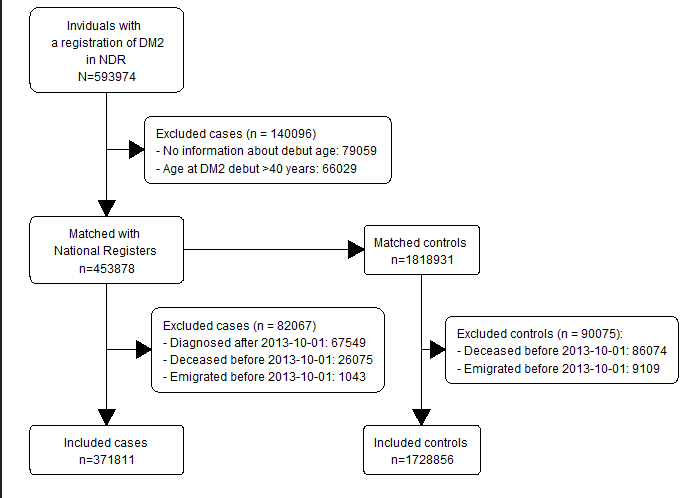


NDR: The Swedish National Diabetes Register. DM: Diabetes Mellitus. SES: Socioeconomic status indicators.
* Diagnosis at the age of 40 or later regardless of diabetes treatment, or treatment with diet or oral agents only regardless of age

**Supplementary figure 5a – i. Non-linear associations between influenza admission and clinical variables in persons with type 2 diabetes**


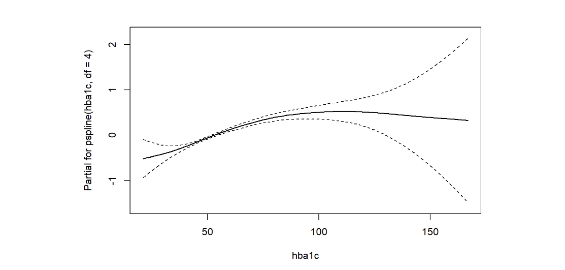

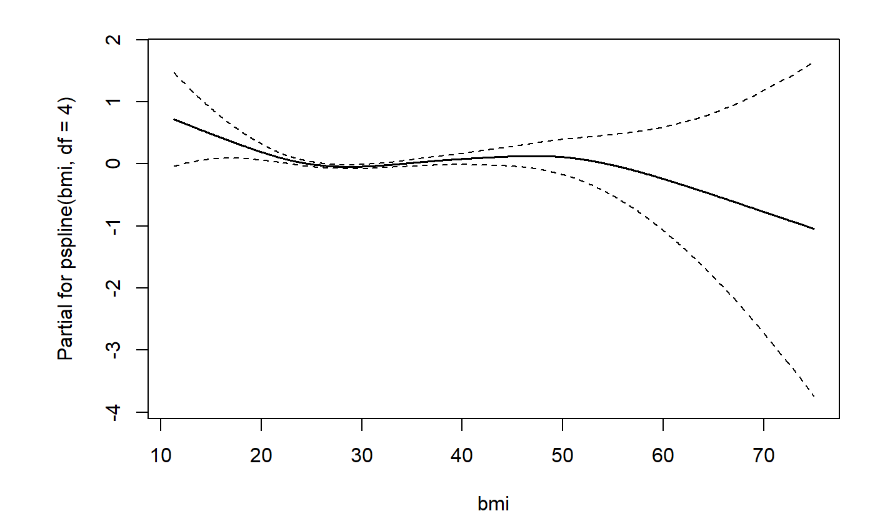


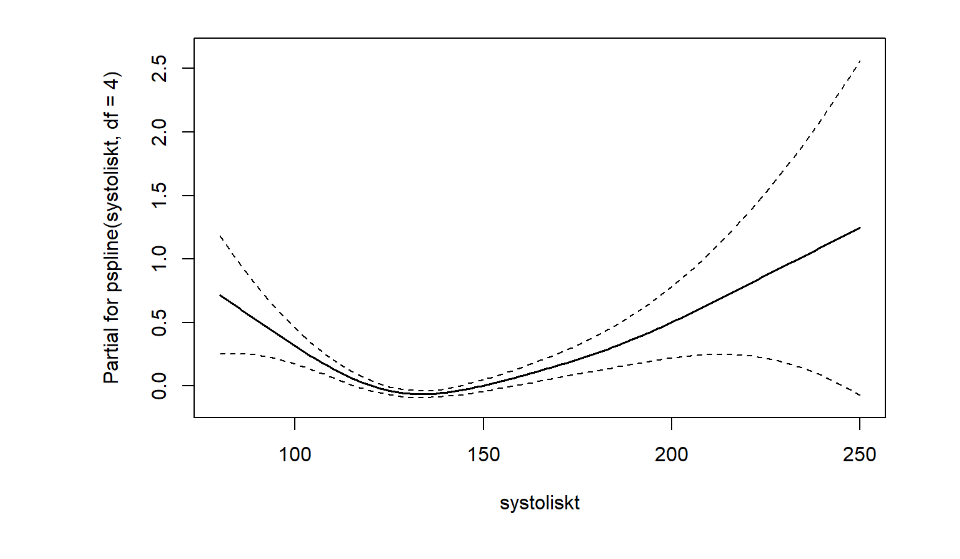

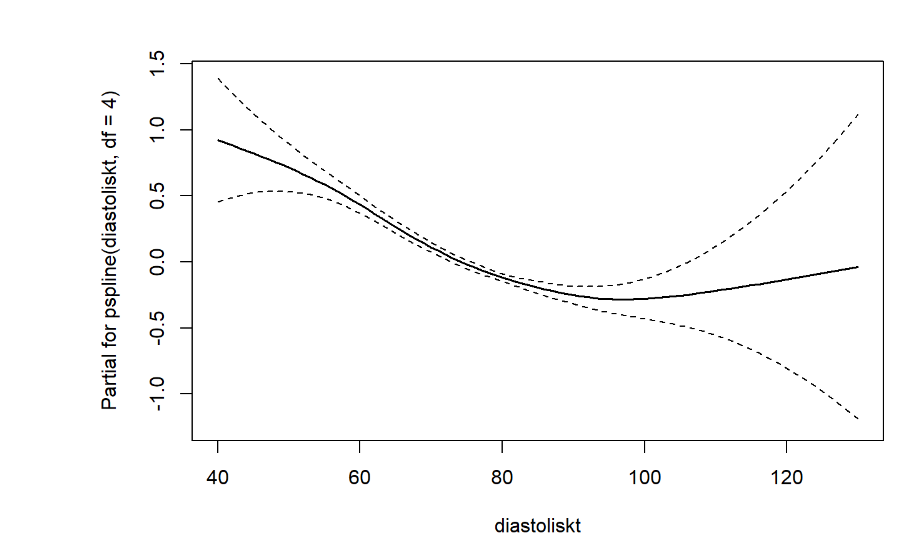

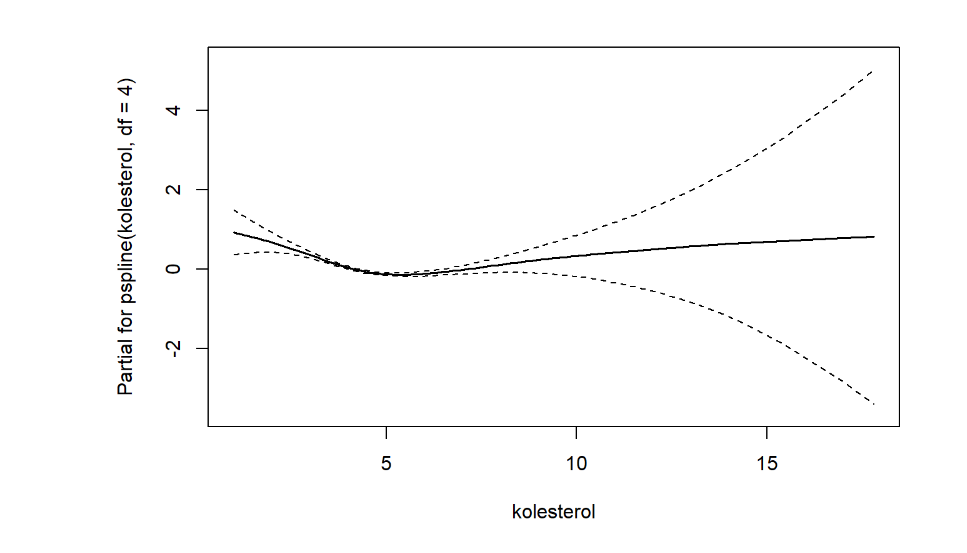


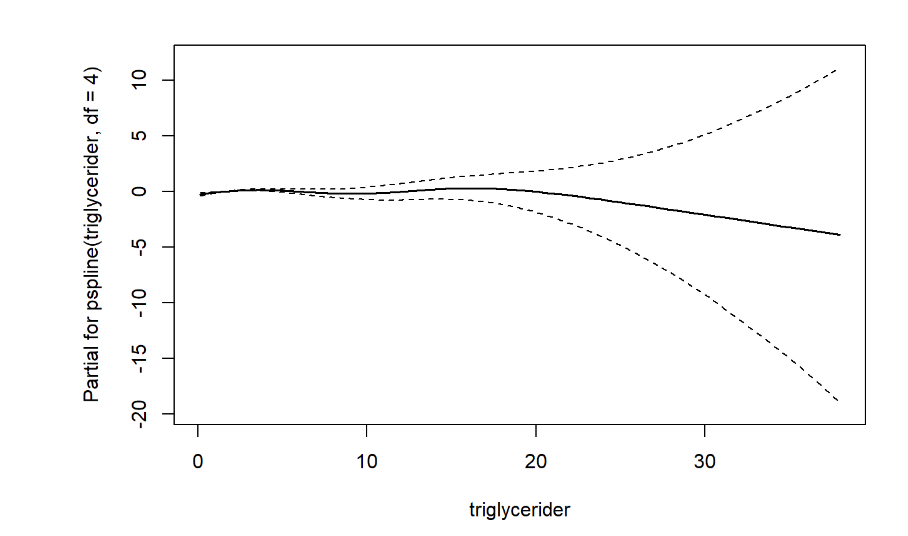

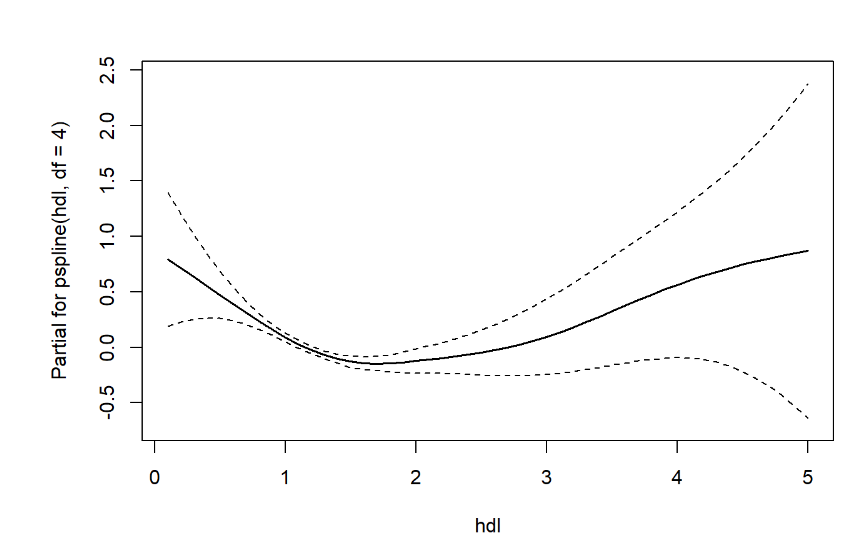

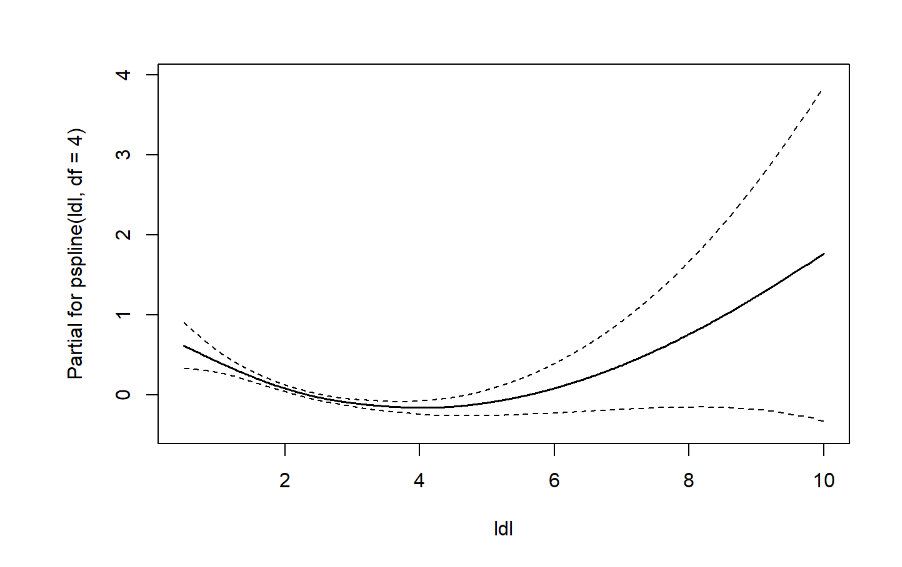

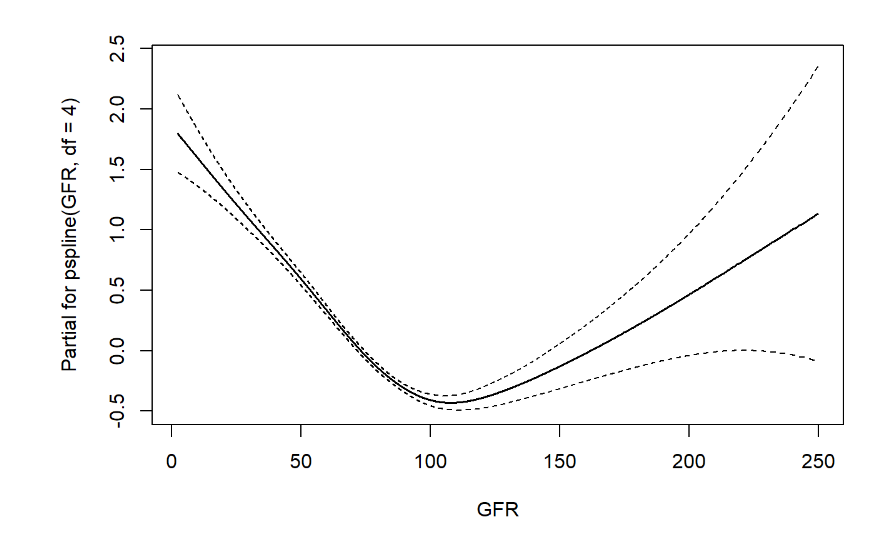


Cox regression with splines (four degrees of freedom) of non-linear associations between numerical clinical variables in persons with type 2 diabetes. Top hand left HbA1c (mmol/mol), second hand left Body Mass Index (kg/m²), third hand left systolic blood pressure (mmHg), forth hand left diastolic blood pressure (mmHg), bottom left total serum cholesterol (mmol/L). Top hand right serum triglycerides (mmol/L), second hand right serum high density lipids (mmol/L), third hand right serum low density lipids (mmol/L), bottom right kidney function (measured by estimated glomerular filtration rate).
