# Supplementary material for: Type 2 diabetes and the risk of hospitalisation and mortality from seasonal influenza: an observational register-based study in Sweden
Source: BMJ Open. 2026 Jun 22;16(6):e106480. doi: 10.1136/bmjopen-2025-106480 (PMC13288867; doi:10.1136/bmjopen-2025-106480)
Supplement: Supplementary file 2 [file bmjopen-2025-106480.reviewer_comments.pdf]

PEER REVIEW HISTORY

BMJ Open publishes all reviews undertaken for accepted manuscripts. Reviewers are asked to complete a checklist review form and are provided with free text boxes to elaborate on their assessment. These free text comments are reproduced below.

ARTICLE DETAILS

Title (Provisional)

Type 2 diabetes and risk of hospitalisation and mortality from seasonal influenza – A observational register-based study from Sweden

Authors

Kjölhede, Elin Allansson; Carlsen, Hanne Krage; Martyn, Oliver; Svensson, Lena; Gisslén, Magnus; Eliasson, Björn; Eeg-Olofsson, Katarina

---

VERSION 1 - REVIEW

**Reviewer**	**1**
**Name**	**Koyama, Alain**
**Affiliation**	**Centers for Disease Control and Prevention**
**Date**	**07-Jul-2025**
**COI**	**None**

The authors report on a study investigating the role of prevalent diabetes in its association with severe outcomes from influenza, finding that adults with diabetes, particularly those with additional comorbidities, are at greater risk of hospitalization or death from influenza, using a large administrative dataset. I have the following comments/suggestions (note the page numbers indicated refer to the numbers at the bottom center of each manuscript page):

Page 6, Methods: "One hospital admission for influenza per season per person" – Does this refer to the first hospital admission for influenza, if there were multiple?

Page 6, Methods: "We collected data on pre-existing diagnoses from Swedish National Patient Register" – Is this derived from self-reported information from clinical visits, diagnosis codes like ICD codes from clinical visits, other information, or some combination of the above?

Page 6, Methods: "We defined influenza season as calendar week 40 thru week 20 the following year in concordance with the vigilance period defined by the Public Health Agency of Sweden." – This time period spans several months from October through May.

Could the risk of influenza hospitalization/mortality vary by month within this time period?

Methods: Were both diabetes cases and controls contributing person time over the entire observation period? Or did they begin to contribute person-time from their index date (first diagnosis of influenza between October 1, 2013 and December 31, 2019) until the end of 2019 or hospitalization or mortality? Given this is a time-to-event analysis, some additional clarity would be useful.

Page 8, Results: "Our closed cohort included 371,811 individuals with type 2 diabetes and 1,728,856 controls" – Since this doesn't add up to exactly a 1:5 ratio, where were some of the patients lost?

Page 12, Discussion: "Compared to Hopkins et al (13)… The studies were conducted at approximately the same time and the same virus variants ought to have circulated in the studied populations" – What are potential explanations for the differences found in this study? There are likely differences attributed to some combination of methodologies, patient populations, healthcare systems, case ascertainment, recording of diagnosis codes, etc

---

Reviewer **2**

Name **Tomic, Dunya**

Affiliation **Monash University, Centre for Occupational and Environmental Health**

Date **05-Jan-2026**

COI **None**

General Comments

This manuscript reports a large, register-based cohort study examining the risk of influenza-related hospitalisation and mortality among adults with type 2 diabetes compared with age- and sex-matched controls in Sweden. The use of national registers, long follow-up, and the ability to examine both absolute and relative risks across clinically relevant subgroups are clear strengths. The focus specifically on type 2 diabetes, rather than diabetes overall, is also a welcome and appropriate distinction. However, the manuscript would benefit from clearer articulation of its aims and more explicit positioning of its contribution relative to existing observational evidence. In addition, several design and analytic choices require clearer justification, and important findings regarding sociodemographic factors and comorbidities are not fully discussed.

Major Comments

1. Conceptual framing and contribution to the literature: The authors appropriately note that type 1 and type 2 diabetes are distinct conditions with potentially different influenza-

related risks, providing a reasonable justification for focusing on type 2 diabetes. However, beyond this distinction, the manuscript does not clearly articulate what gap in the literature is being addressed. While analyses of sociodemographic characteristics and comorbidities are included, there is limited justification for why these factors were prioritised, which dimensions were considered most relevant, and how this extends prior observational evidence. Clearer framing of the study's intended contribution would strengthen the paper.

2. Clarity and alignment of aims and outcomes: The study aims, particularly in the abstract, are somewhat imprecise. The term "background factors" is not clearly defined, and the second aim refers broadly to "clinical variables" without explicitly stating that these analyses are conducted within the type 2 diabetes group. In addition, the "Primary and secondary outcomes" section of the abstract does not clearly state the outcomes of interest, instead focusing on cohort composition and follow-up. Greater precision and consistency across the abstract, introduction, methods, and conclusions would improve readability and transparency.

3. Justification of design and analytic choices: Several methodological decisions would benefit from clearer justification. In particular, the rationale for the definition of the influenza season (calendar week 40 to week 20) should be explicitly stated, ideally with reference to Swedish surveillance practices. In addition, the choice to use matched controls rather than the full population as a comparison group warrants explanation, especially given the availability of national population registers.

4. Interpretation of sociodemographic and comorbidity findings: Although the analyses demonstrate meaningful variation in both absolute and relative risk by age, comorbidity burden, and socioeconomic characteristics, these findings are not explored in depth in the discussion. In particular, the higher relative risks observed among younger individuals with type 2 diabetes, and the attenuation of risk after adjustment for socioeconomic factors and comorbidities, warrant more explicit interpretation. Greater engagement with which factors appear to drive risk differences, and how these findings align with previous studies, would strengthen the contribution.

5. Interpretation and implications given unmeasured vaccination status: The absence of individual-level influenza vaccination data is an important limitation given its likely association with diabetes status, comorbidity burden, and socioeconomic position. While this limitation is acknowledged, greater emphasis is needed on how differential vaccination uptake may influence the observed associations and constrain causal interpretation. Conclusions and recommendations related to vaccination and glycaemic control should be framed with appropriate caution.

Minor Comments

1. The Introduction cites an older edition of the IDF Diabetes Atlas and should be updated to the most recent (11th) edition.

2. The phrase “during the same period” when referring to diabetes prevalence is unclear, as the cited prevalence estimates appear to relate to a single point in time.

3. Some background content on influenza (e.g., circulation in the environment, most infections being mild) could be shortened to improve focus and flow.

4. The 28-day definition of influenza-related mortality should be briefly justified or referenced.

5. There are several grammatical and typographical errors, particularly in later sections of the manuscript, which will require careful language editing.

VERSION 1 - AUTHOR RESPONSE

Reviewer: 1
Dr. Alain Koyama, Centers for Disease Control and Prevention
Comments to the Author:
The authors report on a study investigating the role of prevalent diabetes in its association with severe outcomes from influenza, finding that adults with diabetes, particularly those with additional comorbidities, are at greater risk of hospitalization or death from influenza, using a large administrative dataset. I have the following comments/suggestions (note the page numbers indicated refer to the numbers at the bottom center of each manuscript page):

Page 6, Methods: “One hospital admission for influenza per season per person” – Does this refer to the first hospital admission for influenza, if there were multiple?
Response from the authors: Yes, the first one was recorded. A clarification has been added in the manuscript.

Page 6, Methods: “We collected data on pre-existing diagnoses from Swedish National Patient Register” – Is this derived from self-reported information from clinical visits, diagnosis codes like ICD codes from clinical visits, other information, or some combination of the above?
Response from the authors: Thanks for the opportunity to clarify. The data are based on ICD codes registered by medical doctors at discharge from hospitalisations or at clinical visits. A clarification has been added to the manuscript.

Page 6, Methods: “We defined influenza season as calendar week 40 thru week 20 the following year in concordance with the vigilance period defined by the Public Health Agency of Sweden.” – This time period spans several months from October through May.
Could the risk of influenza hospitalization/mortality vary by month within this time period?
Response from the authors: Thank you for your question. Yes, we have data showing that peak hospitalisation numbers almost always occurred between the end of January and the beginning of March but further analysis of monthly data was not included in this study as our aim was to focus on differences in hospitalization and mortality rates compared to healthy controls over several seasons.

Methods: Were both diabetes cases and controls contributing person time over the entire observation period? Or did they begin to contribute person-time from their index date (first diagnosis of influenza between October 1, 2013 and December 31, 2019) until the end of 2019 or

hospitalization or mortality? Given this is a time-to-event analysis, some additional clarity would be useful.

Response from the authors: Thank you for the opportunity to clarify. The exposure time began at the baseline date October 1 2013, all cases had a diagnosis of type 2 diabetes at that date, and all controls were free of type 2 diabetes on that date.

Page 8, Results: "Our closed cohort included 371,811 individuals with type 2 diabetes and 1,728,856 controls" – Since this doesn't add up to exactly a 1:5 ratio, where were some of the patients lost?
Response from the authors: The controls were matched with their cases at Statistics Sweden, based on their information on sex, age, and residence county at the beginning of the year 2012.

In the data processing for the current study, we identified individuals with a type 2 diabetes diagnosis before October 1, 2013, and selected their matched controls.

In both cases and controls, individuals emigrate or are deceased (n = 6046 DM2 cases, and n= 90075 controls) in the time from the matching date to the formal start date of the study October 1, 2013. However, in this time from matching to start of the analysis period, the rate of immigration is higher in controls vs DM2 cases (0.6% vs 0.3%). More data on this is available in supplementary figure 4.

Page 12, Discussion: "Compared to Hopkins et al (13)… The studies were conducted at approximately the same time and the same virus variants ought to have circulated in the studied populations" – What are potential explanations for the differences found in this study? There are likely differences attributed to some combination of methodologies, patient populations, healthcare systems, case ascertainment, recording of diagnosis codes, etc.
Response from the authors: Thank you for this possibility to clarify. We have added some possible explanations in the discussion.

Reviewer: 2
Dr. Dunya Tomic, Monash University
Comments to the Author:
General Comments

This manuscript reports a large, register-based cohort study examining the risk of influenza-related hospitalisation and mortality among adults with type 2 diabetes compared with age- and sex-matched controls in Sweden. The use of national registers, long follow-up, and the ability to examine both absolute and relative risks across clinically relevant subgroups are clear strengths. The focus specifically on type 2 diabetes, rather than diabetes overall, is also a welcome and appropriate distinction. However, the manuscript would benefit from clearer articulation of its aims and more explicit positioning of its contribution relative to existing observational evidence. In addition, several design and analytic choices require clearer justification, and important findings regarding sociodemographic factors and comorbidities are not fully discussed.

Major Comments

1. Conceptual framing and contribution to literature: The authors appropriately note that type 1 and type 2 diabetes are distinct conditions with potentially different influenza-related risks, providing a reasonable justification for focusing on type 2 diabetes. However, beyond this distinction, the

manuscript does not clearly articulate what gap in the literature is being addressed. While analyses of sociodemographic characteristics and comorbidities are included, there is limited justification for why these factors were prioritised, which dimensions were considered most relevant, and how this extends prior observational evidence. Clearer framing of the study's intended contribution would strengthen the paper.
Response from the authors: Thank you for the possibility to improve the manuscript. We have tried to clarify and explain our study design choices and have added information in the manuscript.

2. Clarity and alignment of aims and outcomes: The study aims, particularly in the abstract, are somewhat imprecise. The term "background factors" is not clearly defined, and the second aim refers broadly to "clinical variables" without explicitly stating that these analyses are conducted within the type 2 diabetes group. In addition, the "Primary and secondary outcomes" section of the abstract does not clearly state the outcomes of interest, instead focusing on cohort composition and follow-up. Greater precision and consistency across the abstract, introduction, methods, and conclusions would improve readability and transparency.
Response from the authors: Thank you for this comment, we have now explained which background factors we chose to focus on and clarified that the analyses of clinical variables were done within the diabetes group. In the abstract we have changed the wording to focus on the outcome variables and which analyses methods we used to study the associations.

3. Justification of design and analytic choices: Several methodological decisions would benefit from clearer justification. In particular, the rationale for the definition of the influenza season (calendar week 40 to week 20) should be explicitly stated, ideally with reference to Swedish surveillance practices. In addition, the choice to use matched controls rather than the full population as a comparison group warrants explanation, especially given the availability of national population registers.
Response from the authors: Thank you for this comment also addressed to some extent by reviewer 1. We have added information on the definition of influenza season in the manuscript to clarify. The rationale for using five reference individuals per person with diabetes instead of the whole population was to have a control population with a similar age, sex and socioeconomic composition as the diabetes population. There is also an ethical and, in some cases, legal issue not to extract more personal data than you need to answer the research questions (data minimization).

4. Interpretation of sociodemographic and comorbidity findings: Although the analyses demonstrate meaningful variation in both absolute and relative risk by age, comorbidity burden, and socioeconomic characteristics, these findings are not explored in depth in the discussion. In particular, the higher relative risks observed among younger individuals with type 2 diabetes, and the attenuation of risk after adjustment for socioeconomic factors and comorbidities, warrant more explicit interpretation. Greater engagement with which factors appear to drive risk differences, and how these findings align with previous studies, would strengthen the contribution.
Response from the authors: Thank you for the comment. We have added to the discussion in accordance with your suggestions and we believe this has strengthened the manuscript.

5. Interpretation and implications given unmeasured vaccination status: The absence of individual-level influenza vaccination data is an important limitation given its likely association with diabetes status, comorbidity burden, and socioeconomic position. While this limitation is acknowledged,

greater emphasis is needed on how differential vaccination uptake may influence the observed associations and constrain causal interpretation. Conclusions and recommendations related to vaccination and glycaemic control should be framed with appropriate caution.
Response from the authors: Thank you for the comments. We have rephrased and added where appropriate to clarify the text.

Minor Comments

1. The Introduction cites an older edition of the IDF Diabetes Atlas and should be updated to the most recent (11th) edition.
Response from the authors: Thank you for spotting this. We have changed the reference. However, number of persons living with type 2 diabetes had to be calculated as the number in the Atlas is given as All persons over the age of 20 with diabetes (588.7 million) and Persons over the age of 20 with type 1 diabetes (1.8 million) are given.

2. The phrase "during the same period" when referring to diabetes prevalence is unclear, as the cited prevalence estimates appear to relate to a single point in time.
Response from the authors: Thank you for spotting this. We have corrected the sentence.

3. Some background content on influenza (e.g., circulation in the environment, most infections being mild) could be shortened to improve focus and flow.
Response from the authors: Thank you for the suggestion. We have changed parts of the introduction.

4. The 28-day definition of influenza-related mortality should be briefly justified or referenced.
Response from the authors: This is a commonly used way of describing mortality in several studies particularly in infectious disease and ICU admissions. If we had chosen a longer period, we would probably have found more mortality cases but the risk over estimating the risk of death attributable to the influenza episode as such would probably be significant. We have clarified this in the manuscript.

5. There are several grammatical and typographical errors, particularly in later sections of the manuscript, which will require careful language editing.
Response from the authors: We have edited the manuscript to improve the readability.

VERSION 2 - REVIEW

Reviewer **2**

Name **Tomic, Dunya**

Affiliation **Monash University, Centre for Occupational and Environmental Health**

Date **29-Apr-2026**

COI

The authors have made meaningful improvements to the manuscript, which is now clearer, better justified, and more coherent than the original. That said, several of my original major concerns remain only partially addressed. Some further targeted revisions would meaningfully strengthen the piece:

1. Positioning relative to existing literature: The introduction still does not clearly articulate what specific gap this study fills. The type 1 vs. type 2 distinction is well motivated, but the contribution beyond this (e.g., that this is the first nationally representative Swedish cohort study of influenza outcomes specifically in type 2 diabetes, or that it provides novel evidence on the role of glycaemic control and renal function) is left implicit. A brief explicit statement of the study's intended contribution at the end of the introduction would orient the reader and strengthen the rationale for the analyses presented.

2. Interpretation of attenuation across adjustment levels: The stepwise adjustment results (HR 1.74 unadjusted, 1.57 fully adjusted for hospitalisation; HR 1.58, 1.44 for mortality) are substantive but not interpreted. The discussion would benefit from explicit engagement with what this attenuation pattern implies (e.g., relative contributions of socioeconomic position versus comorbidity burden in explaining the excess risk, and what the residual ~40-60% relative excess after full adjustment suggests about diabetes-specific mechanisms). This is directly relevant to the clinical and public health implications drawn in the conclusion.

3. Sociodemographic findings: Although baseline differences in income, education, and country of birth between the diabetes and control groups are evident in Table 1, and although these variables are included in the adjustment models, the discussion does not engage with the sociodemographic dimension. Given the policy relevance of identifying socially patterned vulnerability to severe influenza outcomes, even brief discussion of how socioeconomic factors appear to contribute to the observed risk gradient would add value.

4. Minor grammatical issues: Some residual grammatical issues remain (e.g., “than to two-fold” on p. 12 should read “than two-fold”; “infections disease” on p. 6 should read “infectious disease”). A final language pass would address these.

VERSION 2 - AUTHOR RESPONSE

Comments to the Author:
The authors have made meaningful improvements to the manuscript, which is now clearer, better justified, and more coherent than the original. That said, several of my original major concerns remain only partially addressed. Some further targeted revisions would meaningfully strengthen the piece:

1. Positioning relative to existing literature: The introduction still does not clearly articulate what specific gap this study fills. The type 1 vs. type 2 distinction is well motivated, but the contribution beyond this (e.g., that this is the first nationally representative Swedish cohort study of influenza

outcomes specifically in type 2 diabetes, or that it provides novel evidence on the role of glycaemic control and renal function) is left implicit. A brief explicit statement of the study's intended contribution at the end of the introduction would orient the reader and strengthen the rationale for the analyses presented.

Response from the authors: Thank you for pointing this out, we have rewritten the aim section of our introduction to clarify this.

2. Interpretation of attenuation across adjustment levels: The stepwise adjustment results (HR 1.74 unadjusted, 1.57 fully adjusted for hospitalisation; HR 1.58, 1.44 for mortality) are substantive but not interpreted. The discussion would benefit from explicit engagement with what this attenuation pattern implies (e.g., relative contributions of socioeconomic position versus comorbidity burden in explaining the excess risk, and what the residual ~40-60% relative excess after full adjustment suggests about diabetes-specific mechanisms). This is directly relevant to the clinical and public health implications drawn in the conclusion.

Response from the authors: Thank you, we have added a section to the discussion about this result.

3. Sociodemographic findings: Although baseline differences in income, education, and country of birth between the diabetes and control groups are evident in Table 1, and although these variables are included in the adjustment models, the discussion does not engage with the sociodemographic dimension. Given the policy relevance of identifying socially patterned vulnerability to severe influenza outcomes, even brief discussion of how socioeconomic factors appear to contribute to the observed risk gradient would add value.

Response from the authors: Thank you for the opportunity to further discuss the sociodemographic differences between the diabetes group and the reference population. We have added a section in the discussion about this.

4. Minor grammatical issues: Some residual grammatical issues remain (e.g., “than to two-fold” on p. 12 should read “than two-fold”; “infections disease” on p. 6 should read “infectious disease”). A final language pass would address these.

Response from the authors: Thank you, we have now corrected the mistakes.

VERSION 3 - REVIEW

Reviewer **2**

Name **Tomic, Dunya**

Affiliation **Monash University, Centre for Occupational and Environmental Health**

Date **19-May-2026**

COI

The authors have addressed the outstanding concerns from the previous round. The new discussion paragraph engages meaningfully with both the attenuation of hazard ratios across adjustment levels and the sociodemographic dimension of the findings, and the residual

grammatical issues previously flagged have been corrected. I am happy to recommend acceptance.

One small note for copy-editing: the new discussion paragraph contains an incomplete sentence that should be addressed before publication - "In the fully adjusted models, a history of heart failure, kidney disease and chronic respiratory disease with negative outcomes from influenza" appears to be missing a verb (presumably "was associated with").
